# Anemia in Kawasaki Disease: Hepcidin as a Potential Biomarker

**DOI:** 10.3390/ijms18040820

**Published:** 2017-04-12

**Authors:** Ying-Hsien Huang, Ho-Chang Kuo

**Affiliations:** 1 Department of Pediatrics, Kaohsiung Chang Gung Memorial Hospital and Chang Gung University College of Medicine, Kaohsiung 833, Taiwan; yhhuang123@yahoo.com.tw; 2 Kawasaki Disease Center, Kaohsiung Chang Gung Memorial Hospital, Kaohsiung 833, Taiwan

**Keywords:** anemia, hepcidin, Kawasaki disease, iron deficiency

## Abstract

Kawasaki disease (KD) is an autoimmune-like disease and acute childhood vasculitis syndrome that affects various systems but has unknown etiology. In addition to the standard diagnostic criteria, anemia is among the most common clinical features of KD patients and is thought to have a more prolonged duration of active inflammation. In 2001, the discovery of a liver-derived peptide hormone known as hepcidin began revolutionizing our understanding of anemia’s relation to a number of inflammatory diseases, including KD. This review focuses on hepcidin-induced iron deficiency’s relation to transient hyposideremia, anemia, and disease outcomes in KD patients, and goes on to suggest possible routes of further study.

## 1. Kawasaki Disease: The Most Common Acute Coronary Vasculitis Disease in Children

Although its etiology is yet unknown, Kawasaki disease (KD) is an acute childhood vasculitis syndrome that affects various systems [1]. The prevalence of KD in children under the age of 5 years is the highest in Japan with 218/10^5^, followed by Taiwan with 66/10^5^, and the lowest (4.7/10^5^) in Europe [2]. The prevalence of KD is more than 10 times higher in Asian children than in European and American children. KD presents as prolonged fever over five days, bulbar conjunctivitis, diffuse mucosal inflammation, unilateral neck nonsuppurative lymphadenopathy, polymorphous skin rashes, and indurative edema of the hands and feet associated with peeling of the fingertips [2]. Vascular involvement of KD occurs in small and medium-sized blood vessels, particularly the coronary arteries [2]. The most severe complications that KD patients experience are coronary artery lesions (CAL), including myocardial infarction and coronary artery aneurysm (CAA); sequelae of the vasculitis with CAA develop in 20% of untreated children [3]. A U.S. multicenter study group found that a single high dose of 2 g/kg intravenous immunoglobulin (IVIG) combined with aspirin can reduce the incidence of aneurysm from 20–25% to 3–5% [4,5]. However, previous studies have failed to determine a pathogen responsible for KD, or the identified pathogen did not agree among studies [6,7]. While the exact etiology of KD remains uncertain, we have reported that KD stimulates the extraordinary upregulation of TLR1, 2, 4, 5, 6, and 9, which correlates with bacteria-related pathogen-associated molecular patterns, except for the activation of TLR3 and 7, which relates to double-stranded RNA and single-stranded viral RNA in the acute stage of KD. [8]. This study’s results support the idea that KD induces a bacterium-like inflammatory disease. Guo et al. reported that KD’s trademark characteristic is an autoimmune-like disease rather than an infectious disease [9]. Furthermore, children with certain single nucleotide polymorphisms of immune genes (ex. *BLK*, *CD40*, *FCGR2A*, *ITPKC* and *IFNG*) are susceptible to triggering over activated inflammatory reactions through certain pathogens with a unique pathogen-associated molecular pattern, which may be KD’s immunopathogenesis [10,11,12,13,14]. Therefore, KD may be attributed to not only genetic susceptibility, but also to environmental factors and host immune response [15,16]. Currently, no biological markers are available to differentially diagnose KD from other febrile diseases. 

## 2. Anemia in Patients with Kawasaki Disease

In addition to standard diagnostic criteria, KD patients may experience a variety of nonspecific clinical features, including uveitis, aseptic meningitis, abdominal pain, gallbladder hydrops, rash at the bacillus Calmette-Guérin inoculation site, impaired liver function, hypoalbuminemia, and anemia [15,17,18,19]. Of these, anemia is the most common clinical feature in KD patients and is thought to have a more prolonged duration of active inflammation [20,21,22,23]. A dataset of 783 people, including 441 patients with KD and 342 febrile controls, demonstrated that hemoglobin level was among seven variables to have the largest differential diagnostic absolute values of coefficients [24]. Furthermore, Lin et al. observed that hemoglobin is a useful marker for differentiating KD shock syndrome from toxic shock syndrome in a pediatric intensive care unit [25]. Although severe hemolytic anemia requiring transfusion is rare, it may be related to IVIG infusion [21,22,26]. The major causes of hemolysis are generally associated with anti-A and anti-B IgM antibodies, as well as the anti-Rh IgG antibody [27]. In fact, the IVIG products that are used today are usually safe and effective; they are composed of at least 98% of IgG and very low titers of anti-A (1:8) and anti-B (1:4) IgM, but no anti-D IgG antibodies [19,27]. Furthermore, Rh negative blood types are much less common in Asian populations (0.3%) than in Caucasian populations (15%). The phenomena and literature regarding hemolysis after IVIG in KD patients may thus be more commonly reported in European ancestry than Asian ancestry. We also found no significant difference in total bilirubin and haptoglobin levels between KD patients before and after being treated with IVIG [19]. Therefore, we assume that a key pathogenic connection can explain the relationship between KD and anemia. 

Inflammation-associated anemia represents a significant, highly prevalent clinical problem [28]. Chronic disease anemia is often observed in various inflammatory states, such as infections, inflammatory disorders, and certain cancers [29,30,31,32]. In 2000, Krause et al. described a peptide that was first referred to as liver-expressed antimicrobial peptide-1, or LEAP-1 and was later called ‘hepcidin’ due to its hepatic expression and antimicrobial activity [33]. Hepcidin is understood to have a crucial function in blocking the following iron flows into plasma: duodenal absorption, release from macrophages, mobilization of stored iron from hepatocytes, and all that being related to the anemia of inflammation [28,34]. Moreover, high fat diet-induced hepcidin expression is associated with steatosis development and hepatocellular iron accumulation [35,36]. Abnormally elevated hepcidin levels have also been observed in anemia associated with such inflammatory disorders as infections [37,38], autoimmune diseases [39,40], critical illnesses [41,42], obesity [43], and acute myocardial infarction [44].

## 3. Hepcidin Expression Is Correlated with Kawasaki Disease Outcomes

We have previously reported that both plasma hepcidin and IL-6 levels were elevated in KD patients before undergoing IVIG therapy than in febrile controls [45]. Following IVIG treatment, both hepcidin and IL-6 levels decreased significantly. Notably, the changes of hepcidin levels after IVIG administration were related to IVIG treatment resistance and CAL formation, which supports the theory that elevated inflammatory markers and IVIG non-responsiveness may be related to CAL development in KD patients [15,45]. 

Previous studies have proven that IVIG can effectively reduce the incidence of CAL [4], but the role and effective dose of aspirin for KD patients remains unclear. Aspirin-related practices have been administered in KD treatment for the past couple of decades, even prior to the administration of IVIG [3]. Furthermore, anemia and overt bleeding are correlated with aspirin use [46]. We reported in one study of a total of 851 KD patients that high-dose aspirin in acute-phase KD does not confer any benefits on disease outcomes and may even be harmful with regard to reducing disease inflammation [47]. Furthermore, this is the first study to show that high-dose aspirin actually results in lower hemoglobin levels and hinders the ability to decrease hepcidin levels after IVIG treatment. Therefore, high-dose aspirin may not be a necessary part of treatment in acute-phase KD. However, additional randomized placebo control studies are required to clarify the function of high-dose aspirin in KD.

## 4. Hepcidin-Induced Iron Deficiency Is Correlated with Transient Hyposideremia and Anemia in KD Patients

Hepcidin is vital in orchestrating both iron metabolism and the pathogenesis of the anemia of inflammation [48,49]. After hepcidin interacts with ferroportin, ferroportin becomes internalized and degraded, ultimately leading to intracellular iron sequestration and decreased iron absorption [50]. Currently, ferroportin is the only known mammalian iron exporter and is vital for transporting iron from one cell type to another [50]. Hepcidin not only controls iron absorption, but also has an effect on iron-restricted erythropoiesis [51]. Furthermore, hepcidin has also been demonstrated to directly influence erythroid precursor proliferation and survival as erythroid colony formation [52], which agrees with the observation of a transient erythroblastopenia in bone marrow aspiration in KD patients [53]. In our previous study, hemoglobin levels continued to decrease significantly following IVIG treatment, indicating that bone marrow suppression in KD patients is not rapidly reversed following IVIG treatment. Compared to 27 age-matched healthy controls, hemoglobin levels increased at three weeks after IVIG treatment, and hemoglobin levels fully recovered at the six-month follow-up in 117 KD patients. Therefore, we suggest that iron supplementation is not necessary for KD patients.

## 5. Additional Studies Regarding Hepcidin in Kawasaki Disease

Macrophages play a vital role in regulating iron homeostasis, which is closely connected to their polarization during innate immunity. Macrophage iron homeostasis is correlated with the functional polarization and plasticity of these cells, with extreme roles during inflammation, immune modulation, and inflammation resolution [54]. According to the Mosser and Edwards model, macrophage phenotypes are categorized based on their functional characteristics with host defense (M1), wound healing (M2a), and immune regulation (M2b/c), with the concept that the “hybrid-type” macrophages phenotype occurs [55]. Polarization characteristics often refer to cytokine profiles that have been extensively studied in KD patients. However, no studies have yet addressed exact macrophage polarization in KD. Since iron is an essential growth factor for most bacteria and parasites, they have developed various mechanisms to separate iron from the host. Doing so makes M1-macrophages a major iron storage site under inflammatory conditions [54]. In contrast, M2-macrophages increase ferroportin to promote iron release [56]. However, little is known about whether iron homeostasis influences the ability of the macrophage polarization program and molecular machinery involved in KD processes.

## 6. Conclusions

**Figure 1:**
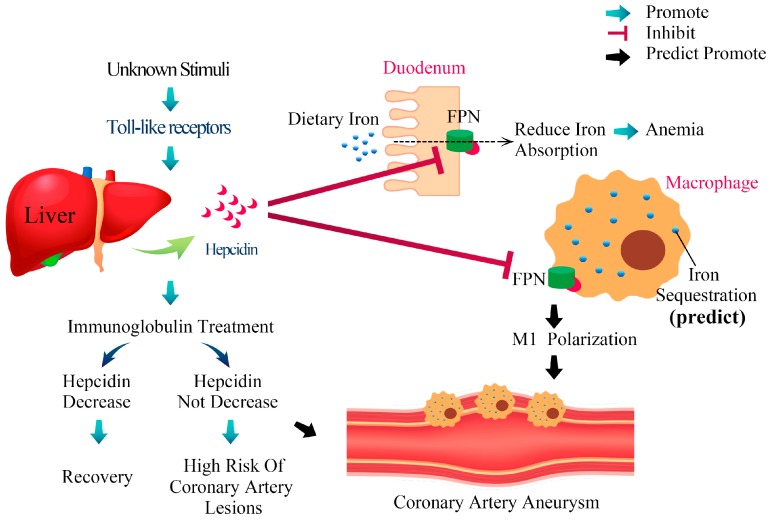
The proposed mechanism of hepcidin-induced transient anemia and coronary artery lesions in patients with Kawasaki disease. While the exact etiology of Kawasaki disease (KD) remains uncertain, we have reported that KD stimulates the extraordinary upregulation of most TLRs that upregulate hepcidin expression. After hepcidin interacts with ferroportin, ferroportin becomes internalized and degraded, ultimately leading to intracellular iron sequestration and decreased iron absorption from duodenum. Hepcidin not only controls iron absorption, but also has a direct inhibitory effect on erythropoiesis, which leads to transient hyposideremia and anemia in KD patients. Following intravenous immunoglobulin (IVIG) treatment, hepcidin levels decrease significantly. Notably, the changes of hepcidin levels after IVIG administration are related to IVIG treatment resistance and coronary artery lesions formation. Macrophage iron homeostasis is correlated with the functional polarization and plasticity of these cells. Doing so makes M1-macrophages a major iron storage site under inflammatory conditions. However, little is known about whether iron homeostasis influences the ability of the macrophage polarization program and molecular machinery involved in KD processes.

Inflammation-induced hepcidin can induce transient hyposideremia, anemia, and disease outcomes in acute-phase KD (Figure 1), and further insightful research is required to better clarify the role of hepcidin in the pathogenesis of KD.

## Abbreviations

CAL	Coronary artery lesions
CAA	Coronary artery aneurysm
IVIG	Intravenous immunoglobulin
KD	Kawasaki disease
